# Anti-Inflammatory, Anti-Bacterial, and Anti-Fungal Activity of Oligomeric Proanthocyanidins and Extracts Obtained from Lignocellulosic Agricultural Waste

**DOI:** 10.3390/molecules28020863

**Published:** 2023-01-15

**Authors:** Anna Andersone, Sarmite Janceva, Liga Lauberte, Anna Ramata-Stunda, Vizma Nikolajeva, Natalija Zaharova, Gints Rieksts, Galina Telysheva

**Affiliations:** 1 Laboratory of Lignin Chemistry, Latvian State Institute of Wood Chemistry, LV-1006 Riga, Latvia; 2 Ekokompozit Ltd., Dzerbenes Street 27, LV-1006 Riga, Latvia; 3 Laboratory of Finished Dosage Forms, Riga Stradins University, LV-1007 Riga, Latvia; 4 Faculty of Biology, University of Latvia, Jelgavas Street 1, LV-1004 Riga, Latvia; 5 Laboratory of Heat and Mass Transfer, The Institute of Physics of University of Latvia, LV-2169 Salaspils, Latvia

**Keywords:** anti-inflammatory, anti-microbial activity, anti-pathogenic activity, anti-fungal, proanthocyanidins, extracts, lignocellulosic biomass, sea buckthorn

## Abstract

It has now been proven that many pathogens that cause infections and inflammation gradually mutate and become resistant to antibiotics. Chemically synthesized drugs treating inflammation most often only affect symptoms, but side effects could lead to the failure of human organs’ functionality. On the other hand, plant-derived natural compounds have a long-term healing effect. It was shown that sea buckthorn (SBT) twigs are a rich source of biologically active compounds, including oligomeric proanthocyanidins (PACs). This study aimed to assess the anti-pathogenic and anti-inflammatory activity of water/ethanol extracts and PACs obtained from the lignocellulosic biomass of eight SBT cultivars. The anti-pathogenic activity of extracts and PACs was studied against pathogenic bacteria *Pseudomonas aeruginosa, Staphylococcus aureus, Escherichia coli, Bacillus cereus* and fungus *Candida albicans* in 96-well plates by the two-fold serial broth microdilution method. The anti-bacterial activity of purified PACs was 4 and 10 times higher than for water and water/ethanol extracts, respectively, but the extracts had higher anti-fungal activity. Purified PACs showed the ability to reduce IL-8 and IL-6 secretion from poly-I:C-stimulated peripheral blood mononuclear cells. For the extracts and PACs of SBT cultivar ‘Maria Bruvele’ in the concentration range 0.0313–4.0 mg/mL, no toxic effect was observed.

## 1. Introduction

Following the global response to SARS-CoV-2, emerging antibiotic resistances were evaluated as a “silent pandemic”, putting the ability to effectively combat prevalent infectious diseases at risk [1]. Estimates from the European Union/European Economic Area (EU/EEA) alone show that each year, more than 670,000 infections occur due to bacteria being resistant to antibiotics, and approximately 33,000 people die as a direct consequence [2]. Infections caused by bacteria forming biofilms are much less susceptible to antibiotics [3,4]. Bacterial infections are a common complication after primary infection with respiratory viruses such as influenza viruses, rhinoviruses, and coronaviruses and are often characterized by severe disease and high mortality [5]. New powerful anti-microbial agents are necessary, but stronger antibiotics of synthetic origin cause more severe side effects and “sweep away” both pathogenic and beneficial microorganisms.

Moreover, one of the side effects of antibiotics overuse is the development of resistant fungal infections. The polymorphic fungus *Candida albicans* is a member of the normal human microbiome, but under certain circumstances, especially among immunocompromised populations, it causes infections that range from superficial infections of the skin to life-threatening systemic infections in the bloodstream or internal organs [6]. *Candida* is the fourth most common cause of hospital-acquired systemic infections in the United States, with crude mortality rates of up to 50% [7]. Treatment for these fungal infections has adverse effects and is slowly becoming obsolete due to varying mutation rates and rising resistance in multiple species. For example, about 7% of all *Candida* blood samples tested at the CDC are resistant to the most common anti-fungal drug fluconazole [8].

Finding natural compounds that would effectively act on bacterial and fungal infections, independently or synergistically with anti-bacterial or anti-fungal treatments, and increasing the body’s own ability to overcome the infection without exerting toxicity on internal organs are challenging emerging tasks today.

Infections can become more dangerous if there is an excessive inflammatory response. Many kinds of cells and active ingredients (cytokines, chemokines, and bioactive amines) participate in inflammation [9]. The accompanying SARS-CoV-2 cytokine storm—a group of related medical conditions in which the immune system produces too many inflammatory signals—can lead to organ failure and death. Inflammation has a protective function, but excess inflammation can induce host tissue damage, chronic diseases, and even cancer [10,11]. Currently, anti-inflammatory drugs are mainly steroidal and non-steroidal drugs, but they have frequent clinical side effects. The development of safer alternatives has attracted widespread attention.

One of the possible solutions is the use of natural molecules with anti-bacterial, anti-fungal, and anti-inflammatory activity. Plant extracts are complex mixtures containing a wide variety of primary and secondary metabolites, and their action may be the result of the synergy of different chemical components. Plant preparations have a number of advantages in particular due to the absence of side effects and a decrease in toxic effects for the body, while they cause complex pharmacological effects.

Plant-derived compounds, including alkaloids, phenolic acids, flavonoids, carotenoids, coumarins, terpenes, proanthocyanidins (PACs), and some primary metabolites (amino acids, peptides, organic acids) exhibit anti-microbial and anti-inflammatory properties [4,5,12,13,14]. Dietary polyphenols such as flavonoids, phenolic acids, and PACs in large quantities in foods of plant origin exhibit many beneficial effects and play an important role in the prevention of chronic and degenerative diseases. These dietary polyphenols are also found in lignocellulosic biomass (twigs, bark) in both deciduous and fruit trees/bush species.

PACs in plants represent the first biochemical defense against external injuries and infections [15,16]. Chemically, PACs are oligomers (degree of polymerization (DP) = 2–5) and polymers (DP > 5) or polymers of monomeric flavan-3-ols produced as an end product of the flavonoid biosynthetic pathway. Studies have demonstrated the biological activities of PACs [17,18,19,20,21].

The most widely studied PACs of grape seeds have been reported to exhibit anti-inflammatory activity by reducing the accumulation of pro-inflammatory cytokines [22], reducing aerobic and anaerobic microorganisms’ colonies in plaque [23] and preventing gastrointestinal bacterial infections [24]. The latest studies showed that cranberry PACs prevent the evolution of resistance to tetracycline in *Escherichia coli* and *Pseudomonas aeruginosa*, rescue antibiotic efficacy against antibiotic-exposed cells, and inhibit biofilm formation [25]. PAC oligomers isolated from peanut skin (*Arachis hypogaea* L., Fabaceae) were reported to have the potential to reduce inflammation and melanogenesis [26]. PACs’ properties are related to their chemical structure, as they have phenolic rings that can bind to a wide range of molecules and act as electron scavengers by capturing ions and radicals [24].

Our preliminary studies of the chemical composition of extracts isolated from the waste biomass of sea buckthorn (SBT) and other wood species (grey alder, black alder, willow, pine) by water and aqueous ethanol solutions showed that the PACs are the dominant polyphenolic compounds in the extracts [27]. In previous research, it was shown that SBT twigs are a valuable and cheap source of PACs, and they have higher anti-microbial activity than extracts by themselves [28]. In a range of European countries such as Latvia, Estonia, Romania, and Germany, as well as in Canada and China where SBT is cultivated on plantations, a large volume of underutilized lignocellulosic biomass waste forms as a result of agrotechnical measures carried out for SBT twice per year, at yearly industrial harvesting of the berries, which includes cutting the whole branch (20% of the berries’ mass) and pruning (90% of SBT plantation biomass every fourth year, almost a full cut of the whole shrub tree) [28].

It is known that PACs can prevent bacteria from attaching to cell or biomaterial surfaces [3,4,29,30] by impairing bacterial motility [5,9,10,11,13,14]. It is also suggested that PACs may inhibit biofilm formation and enhance the effect of gentamicin against *P. aeruginosa*. There is evidence that in the presence of PACs, the acquisition of resistance in *E. coli* and *P. aeruginosa* after treatment with tetracycline is completely stopped. Thus, PACs have the ability to interfere with the mechanisms of intrinsic resistance, thus suppressing the typically inevitable long-term evolution of acquired antibiotic resistance [15]. Due to their antioxidant and anti-inflammatory properties, PACs reduce inflammation in an animal model of gastric and colonic inflammation [31].

Herbal products were discarded from conventional medical use in the mid-20th century, not necessarily because they were ineffective but because they were not as economically profitable as the newer synthetic drugs. In spite of this, the global herbal medicine market was valued at USD 151.91 billion in 2021 and projected to grow from USD 165.66 billion in 2022 to USD 347.50 billion by 2029 [32].

The aim of this study was the assessment the role of PACs in the anti-inflammatory and anti-microbial activity of the ethanol/water extracts obtained from lignocellulosic agricultural waste of eight SBT cultivars: ‘Maria Bruvele’, ‘Tarmo’, ‘Tatiana’, ‘Duet’, ‘Leikora’, ‘Clara’, ‘Otto’, and ‘Botanicheskaya Lubitelskaya’. The anti-inflammatory effect was evaluated by the reduction in IL-8 and IL-6 secretion, one of the major mediators of the inflammatory response, an early-phase biomarker, in the presence of extracts and PACs.

The anti-bacterial activity was tested toward Gram-positive bacteria, *Staphylococcus aureus* and *Bacillus cereus*, as well as Gram-negative bacteria, *Pseudomonas aeruginosa* and *Escherichia coli*, and fungus *Candida albicans*. *S. aureus* is a leading pathogen associated with a number of diseases, including osteomyelitis, pneumonia, endocarditis, and septicemia. *B. cereus* produces toxins, causing two types of gastrointestinal illness: emetic (vomiting) syndrome and diarrheal syndrome. Multidrug-resistant *P. aeruginosa* is the one that most often causes infections in humans and causes infections and deaths among hospitalized patients [33]. Several strains of *E. coli* are enteric pathogens associated with hemorrhagic colitis and the development of the life-threatening condition hemolytic uremic syndrome (HUS) [34].

## 2. Results and Discussion

### 2.1. Chemical Composition

**Figure 1:**
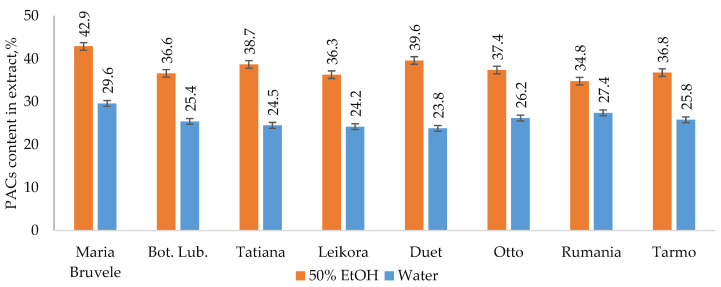
PAC content in extract isolated by 50% EtOH or water from eight SBT cultivars’ twigs.

The results of extraction by 50% ethanol (50% EtOH) and water show that the yields of hydrophilic extract substances of all SBT twigs’ biomass under study varied from 19% to 29%. UHPLC-ESI-MS/MS profile of twigs’ extracts from all SBT cultivars contained the complex phenolic fingerprint with different phenolic compounds identified, comprising oligomeric and monomeric flavonoids. Oligomeric flavonoids—PACs (mainly B-type PACs)—were the dominant polyphenols in the extract’s composition. The PAC content in 50% EtOH extracts was higher than that found in water extracts and ranged from 34.8 to 42.9% in 50% EtOH extracts and from 23.8 to 29.6% in water extracts of the cultivars tested (Figure 1).

**Figure 2:**
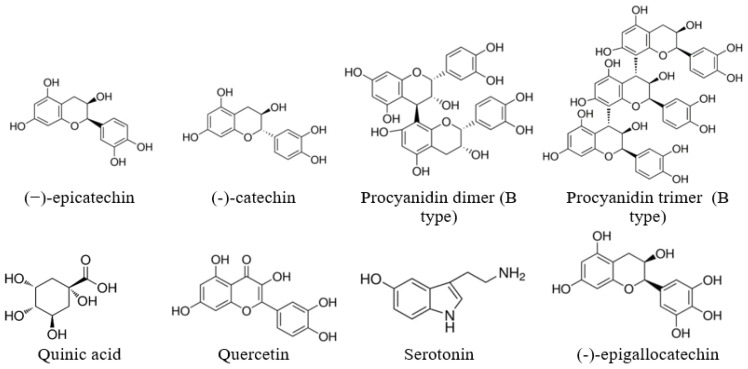
Some of the identified components of the extracts.

For the extracts obtained using the same solvent, the PAC values were close to each other for all SBT cultivars growing on the same plantation, which indicates the similarity of their ability to synthesize secondary metabolites. SBT extracts contain not only oligomeric PACs, but also other polyphenolic compounds such as quercetin (16), quinic acid (2), and gallocatechin or its isomer epigallocatechin (4), which have excellent documented anti-bacterial activity against *Staphylococcus aureus, Bacillus subtilis*, and *Escherichia coli* [35,36]. Polar triterpenoids are also present in the composition of the extracts, which for the extracts from the other plants, have shown strong anti-inflammatory effects [37]. The list of identified components is shown in Table 1, and the structures of some of the identified components in Figure 2.

**Figure 3:**
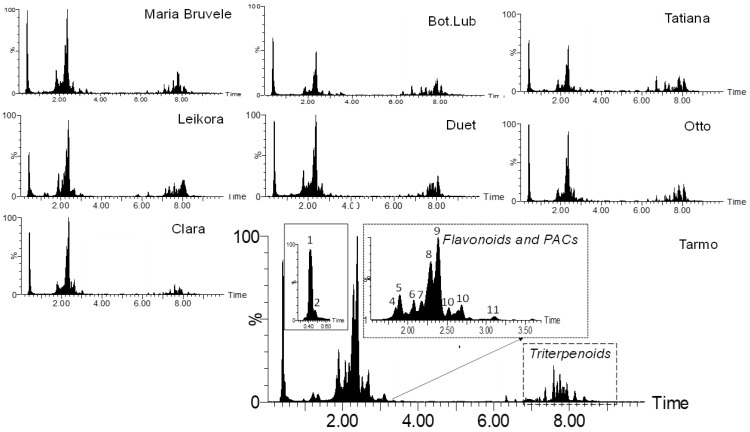
Comparison of the chemical composition of the sea buckthorn 50% EtOH extracts by UHPLC-TOF/MS chromatograms.

**Figure 4:**
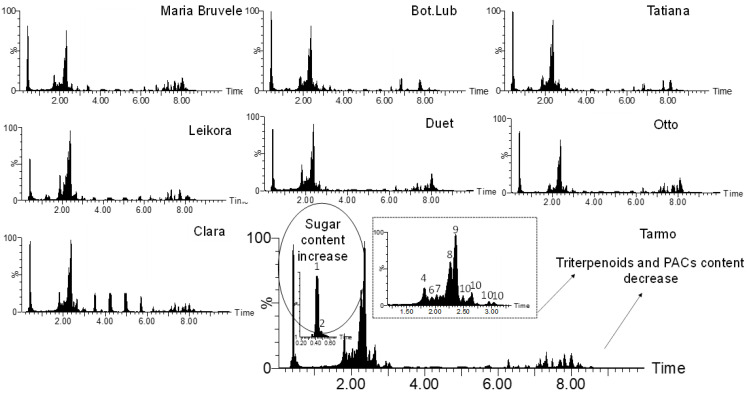
Comparison of the chemical composition of the sea buckthorn water extracts by UHPLC-TOF/MS chromatograms.

When comparing the chemical composition of 50% EtOH and water extracts of eight varieties of SBT, the following changes were noted: the decrease in the content of monomeric and oligomeric flavonoids (Figure 1, Figure 3, and Figure 4) and triterpenoids in water extracts (Figure 4), as well as the increase in the content of carbohydrates (in free and glycosidic form), which can adversely affect biological activity. The most abundant monosaccharides in the water extract of all cultivars were sucrose, fructose, and glucose.

### 2.2. Anti-Bacterial and Anti-Fungal Activity

The anti-microbial activity of extracts obtained by extraction with water and 50% EtOH from eight SBT cultivars’ twigs is shown in Table 2.

The results showed that all the extracts inhibit the growth of Gram-positive and Gram-negative bacteria as well as pathogenic fungus. Moreover, the inhibitory activity of almost all the extracts against *S. aureus* and *E. coli* is higher than the data found in the literature for well-known natural anti-bacterial extracts of *Echinacea purpurea* and *Arctium lappa* (MIC: 2.93 mg/mL, MBC: 5.86 mg/mL, for both plants), and for some of the extracts (‘Otto’ and ‘Tarmo’), the anti-fungal activity against *Candida albicans* is similar to the mentioned plant extracts (MIC: 5.86 mg/mL, MFC: 11.72 mg/mL, for both *Echinacea purpurea* and *Arctium lappa*) [38]. The MIC of the extracts under study was also lower than that of garlic (MIC of hybrid garlic against *S. aureus* started at a concentration of 5.0 mg/mL for water extracts and at 10 mg/mL for ethanol extract) [39].

For most of the extracts, MICs/MBCs against *E. coli*, *P. aeruginosa*, and *S. aureus* are less than 2 mg/mL, which accounts for a high anti-bacterial activity for the natural compounds in the literature [40]. This allows the consideration of the SBT extracts’ prospective for natural anti-bacterial preparations.

The extracts isolated with 50% EtOH were more effective than water extracts; this could be due to the higher content of PACs in the extracts (Figure 4). In relation to *E. coli*, extracts isolated from SBT twigs of cultivars ‘Duet’, ‘Otto’, ‘Clara’, and ‘Maria Bruvele’ were more active than those from other cultivars. However, 50% EtOH extract from ‘Clara’ twigs showed weaker activity against *S. aureus* bacteria (MIC: 1.56 mg/mL). The MIC concentration in relation to *P. aeruginosa* was the same for all extracts (0.78 mg/mL), except for the extract from ‘Tatyana’ twigs (4 times weaker—3.13 mg/mL) and ‘Maria Bruvele’ twigs (2 times more effective—0.39 mg/mL). In the inhibition of *B. cereus*, the extract from ‘Bot. Lub.’ twigs by 50% EtOH was two times weaker (0.78 mg/mL) than other 50% EtOH extracts. In relation to *C. albicans*, extracts from ‘Maria Bruvele’, ‘Bot. Lub.’, and ‘Tatiana’ were more effective compared to other SBT cultivars.

The lowest MIC values were found for water extracts isolated from the twigs of ‘Duet’ (12.5 mg/mL against *E. coli*; 12.5 mg/mL against *S. aureus*), ‘Leikora’ (12.5 mg/mL to *C. albicans*), and ‘Otto’ (6.25 mg/mL against *P. aeruginosa* and *S. aureus*). ‘Clara’ and ‘Tarmo’ water extracts were also less effective in inhibiting *C.albicans*. Among all extracts, 50% EtOH extract isolated from ‘Maria Bruvele’ twigs showed the highest biological effect on all types of pathogenic bacteria (MIC: 0.2–0.39 mg/mL). The following tendency of microbial sensitivity was observed: *E. coli* = *S. aureus* > *P. aeruginosa* = *B. cereus*. For a water extract, the tendency of bacteria sensitivity was very similar: *E. coli* = *S. aureus* = *P. aeruginosa* > *B. cereus* (Table 2).

The minimum bactericidal/fungicidal concentration (MBC/MFC) is the lowest concentration of an anti-bacterial/anti-fungal sample required to kill bacteria/fungi over a fixed period under a specific set of conditions. The MFC determination could be useful in severe fungal infections in immunocompromised patients. According to the summarized results presented in Table 2, the extracts isolated by 50% EtOH from all SBT cultivars were the most active against such strains as *E. coli*, *P. aeruginosa*, and *S. aureus*.

Comparing the MBC value of 50% EtOH extracts with water extracts, water extracts were weaker, especially those of cultivars ‘Bot.Lub.’, ‘Duet’, and ‘Otto’. Quantitative analysis of water extracts showed the highest content of sugars (sucrose, glucose, fructose) and polyphenolic glycosides (quercetin-3-O-rutinoside), which could be the reason for the higher MBC of the aforementioned cultivars. The MBC of water extract from the ‘Maria Bruvele’ cultivar in relation to *E. coli* was 2 times higher, in relation to *P. aeruginosa* it was 4 times higher, and in relation to *S. aureus* it was 2 times higher than for 50% EtOH extract; the MBC against *B. cereus* and MFC against *C. albicans* were ≥50 mg/mL for both water and 50% EtOH extract, which indicates that a higher concentration is probably needed.

**Figure 5:**
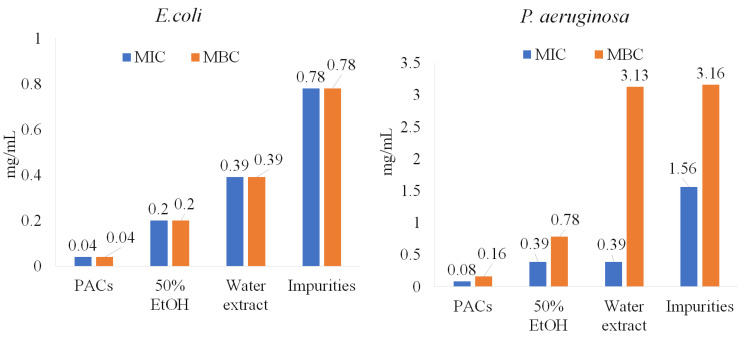
PACs’ role in anti-bacterial activity against *E. coli* and *P. aeruginosa*.

As is shown in Figure 1, PACs are the dominant polyphenolic compounds in all the extracts. PACs were isolated and further tested on pathogenic bacteria under study (*E. coli*, *P. aeruginosa*, *S. aureus*, and *B. cereus*). The purity of PACs from the ‘Maria Bruvele’ 50% EtOH extract was 92% (determined by the butanol-acid method). In relation to *E. coli*, the isolated PACs were 5 times more effective than the 50% EtOH extract and nearly 10 times more effective than the aqueous extract (Figure 5).

**Figure 6:**
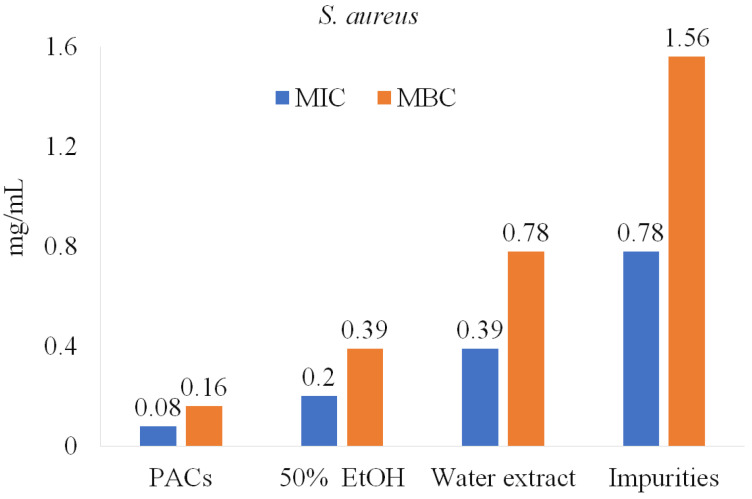
PACs’ role in anti-bacterial activity against *S. aureus*.

Similar results were observed for *P. aeruginosa*, *S. aureus*, and *B. cereus* (Figure 6). Impurities were also tested and showed lower anti-bacterial activity potency, suggesting that PACs have a key role in inhibiting pathogenic bacteria.

Oral candidiasis is a common fungal disease caused mainly by *C. albicans*. When comparing the anti-fungal activity of three samples (50% EtOH, water extract, and PACs of ‘Maria Bruvele’), isolated PACs were weaker at the initial stage. The following MIC tendency was observed: 50% ethanol extract (0.20 mg/mL) > water extract (0.39 mg/mL) > PACs (1.25 mg/mL) (Table 3).

It is assumed that at the initial stage of anti-fungal activity, the low-molecular-weight polyphenolic compounds listed in Table 1 have a synergistic effect with PACs, thereby improving the MIC value. However, only purified PACs have fungicidal activity (MBC = 1.25 mg/mL). The complex chemical structure of PACs and their composition, which includes a dimer, a trimer, a tetramer, etc., have a synergistic anti-microbial effect and will not allow the fungus to develop resistance to them.

### 2.3. Cytotoxicity Assessment

**Figure 7:**
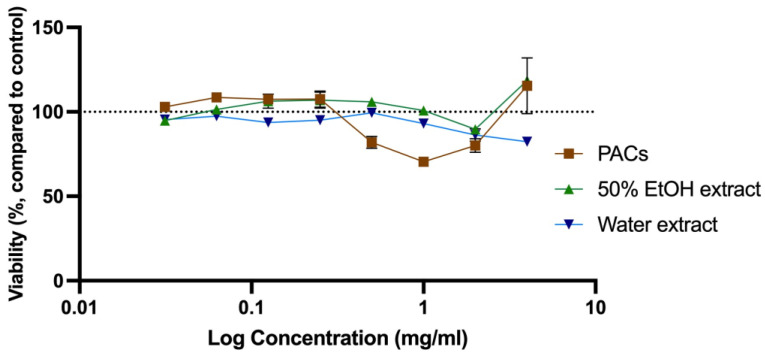
Cytotoxicity of SBT extracts evaluated as viability changes in neutral red uptake test in Balb/c 3T3 cell culture (n = 3). The dotted line corresponds to the control level.

The cytotoxicity was evaluated to determine the toxic concentration of the extracts and PACs and compared with the anti-microbial concentration observed (MIC) for further analysis of their application in anti-microbial therapy [28,41]. Cytotoxicity of all SBT extracts was tested at a concentration range of 0.0313–4.0 mg/mL. Water extracts were slightly more cytotoxic than ethanol extracts. An extract at a specific concentration was considered to be cytotoxic if the cell viability was reduced by more than 20%. Cytotoxic concentrations of 50% EtOH and water extracts from ‘Maria Bruvele’, as well as isolated PACs from ‘Maria Bruvele’ extract, did not exceed the concentrations needed to inhibit the growth of the tested microorganisms. Samples tested in the concentration range of 0.0313–4.0 mg/mL did not have cytotoxicity except the PAC sample that, at a concentration of 1 mg/mL, reduced cell viability by 29.56% (Figure 7).

### 2.4. Hemolysis

**Figure 8:**
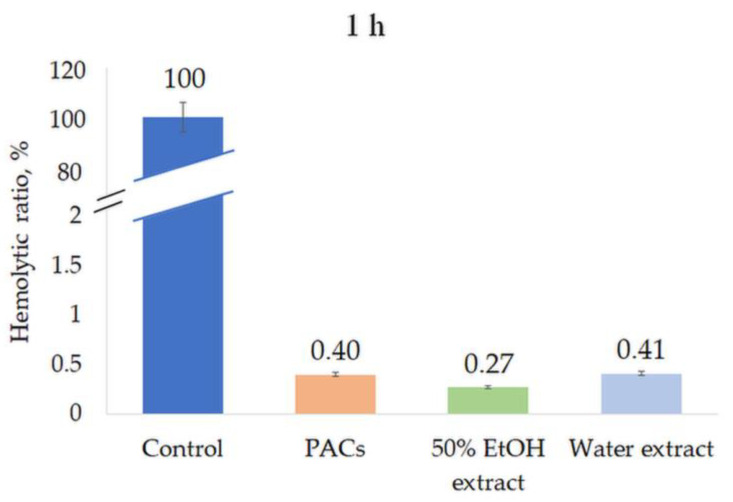
Hemolytic ratio (%) of ‘Maria Bruvele’ twigs’ samples in fresh human blood hemolysis test after 1 h incubation. Control—deionized water, n = 3.

**Figure 9:**
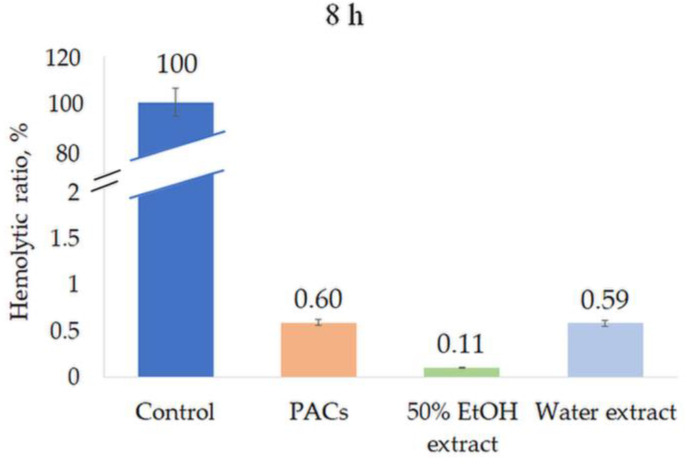
Hemolytic ratio (%) of ‘Maria Bruvele’ twigs’ samples in fresh human blood hemolysis test after 8 h incubation. Control—deionized water, n = 3.

All extracts were tested for their hemolytic activity at a concentration of 0.5 mg/mL. None of the extracts induced hemolysis after 1 h or 8 h incubation, indicating the high biocompatibility and safety of the extracts (Figure 8 and Figure 9).

### 2.5. Immunomodulating Activity

**Figure 10:**
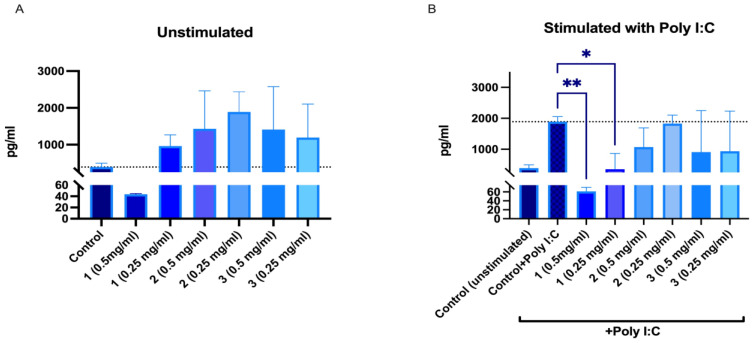
Changes in IL-8 secretion from unstimulated (**A**) and poly-I:C-stimulated (**B**) human peripheral blood mononuclear cells after 24 h incubation with SBT samples: 1—PACs, 2—50% EtOH extract, 3—water extract. * *p* < 0.05, ** *p* < 0.01 one-way ANOVA, n = 3. The dotted line corresponds to the control level.

IL-6 and IL-8 secretion in human peripheral blood mononuclear cells (PBMNCs) after 24 h incubation with SBT samples was investigated. Although the mouse blood macrophage RAW264.7 cell model is popular for screening immunomodulating activities, we chose human PBMNCs as a more relevant model. There have been studies with known immunomodulators that showed differences in how RAW264.7 and human PBMNCs respond. Compounds and extracts that might have immunomodulatory activity in humans might be missed in rodent cell models as they may not always mimic the responses of human immune cells [42]. It should be noted that PBMNCs are a heterogenous cell population that, in the case of immunomodulation studies, provides additional benefits in assessing overall effects on immune cells. According to the data obtained, 50% EtOH and water extracts from ‘Maria Bruvele’ increased the IL-8 secretion at both tested concentrations. PACs at a concentration of 0.5 mg/mL reduced IL-8 secretion in unstimulated PBMNCs and significantly reduced IL-8 secretion in poly-I:C-stimulated PBMNCs (Figure 10).

Polyinosinic:polycytidylic acid (poly I:C) mimics viral double-stranded RNA and binds TRL3 receptors of human cells, thus mimicking inflammation related to viral infections. All ‘Maria Bruvele’ samples (PACs, 50% EtOH extract, and water extract) reduced secretion of IL-8 in the presence of poly I:C. Results indicate the ability of plant PACs and PACs containing extracts to reduce inflammation related to viral infections (Figure 10). The best results were observed for isolated PACs from ‘Maria Bruvele’ 50% EtOH extract.

**Figure 11:**
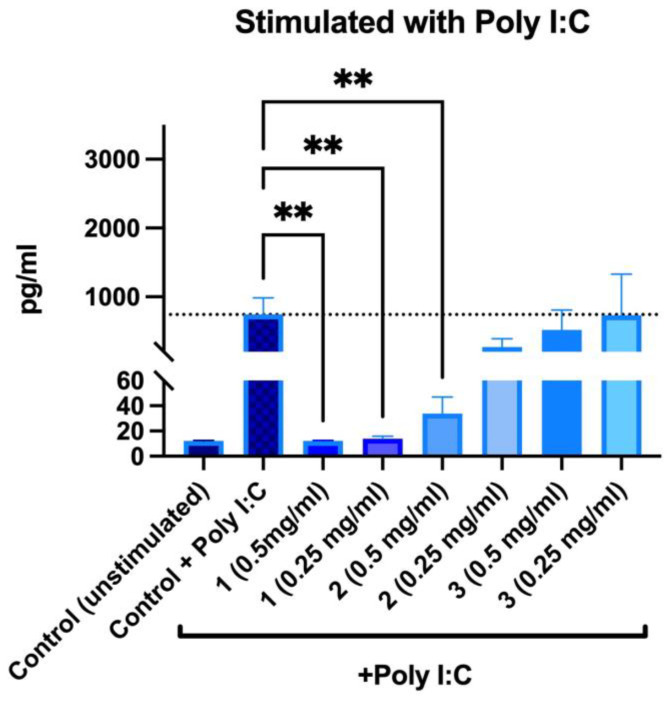
Changes in IL-6 secretion in stimulated peripheral blood mononuclear cells after 24 h incubations: 1—PACs, 2—50% EtOH extract, 3—water extract. ** *p* < 0.01 one-way ANOVA, n = 3. The dotted line corresponds to the control level.

Without poly I:C stimulation, no increase in IL-6 secretion was observed after incubation with the samples. When samples and poly I:C were added to PBMNCs simultaneously, it was observed that PACs and ethanolic extracts significantly reduced secretion of IL-6 (Figure 11).

In presence of PACs, IL-6 secretion was reduced to an unstimulated control level; furthermore, no differences were observed between both tested concentrations. For 50% EtOH extracts, the inhibitory effect was concentration-dependent—0.5 mg/mL reduced IL-6 secretion by 95.43%, whereas 0.25 mg/mL reduced it by 63.75%. Water extracts did not reduce poly-I:C-induced IL-6 secretion. Overall, our findings are consistent with other biomass studies where the effects of PACs on IL-6 and IL-8 secretion have been described in inflammation models [43,44,45].

To our knowledge, there are just a few studies describing the effects of PACs on poly-I:C-induced inflammation in vitro and no previous studies on PACs specifically isolated from SBT.

## 3. Materials and Methods

### 3.1. Materials

#### 3.1.1. SBT Biomass

The twigs of eight sea buckthorn cultivars (*Hippopae rhamnoides* ‘Leikora’, ‘Otto’, ‘Clara’, ‘Duet’, ‘Tamo’, ‘Tatiana’, ‘Maria Bruvele’, and ‘Botanisheskaya Lubitelskaya’) and leaves of ‘Maria Bruvere’ were collected from the sea buckthorn (SBT) plantation area in Latvia in the late summer of 2020. The twigs and leaves were dried at room temperature and ground with a knife mill (Cutting Mill SM100, Retsch, Haan, Germany). The particle size of the grounded SBT twigs was between 1 and 4 mm, and the leaves were between 0.5 and 1 mm.

#### 3.1.2. Chemicals

Procyanidin B2 (≥90% HPLC) analytical standards were purchased from Sigma-Aldrich (Merck KGaA, Darmstadt, Germany), LC-MS hyper grade acetonitrile from Merck (LiChrosolv^®^, Merck KGaA, Darmstadt, Germany), and formic acid (HiperSOLV Chromanorm) from VWR Chemicals (Radnor, PA, USA). Milli-Q Type 1 ultrapure water (suitable for chromatography and other advanced analytical techniques) was used for sample preparation as well as the mobile phase.

Reagents including FeNH_4_(SO_4_)_2_∙12 H_2_O, *n*-butanol (purity ≥ 99.4%), and crosslinked dextran-based resin Sephadex LH-20 were purchased from Aldrich Sigma (Merck KGaA, Darmstadt, Germany).

### 3.2. Methods

#### 3.2.1. PAC-Rich Extract Isolation from SBT Biomass

Extracts were isolated by the convective extraction of SBT biomass at 60 °C for 30 min using the following solvents: distilled water or aqueous ethanol (1:1, *v*/*v*). The extracts were freeze-dried to yield a brown solid. The yield of the extracts is presented as a percentage based on the oven-dried (o.d.) biomass.

#### 3.2.2. Determination of PAC Content in the Extract

PAC content in the extracts was measured by the butanol–HCl method [46] using procyanidin dimer B2 as a reference compound. Amounts of 6 mL of acid butanol (5% (*v*/*v*) concentrated HCl in n-butanol) and 0.2 mL of iron reagent (*w*/*v*) (FeNH_4_(SO_4_)_2_∙12 H_2_O in 2 N HCl) were added to 1 mL of the extract aliquots whilst stirring the tube without heating and allowing it to be heated in a water bath at 80 °C for 50 min. After 50 min, the absorbance of the mixture was measured against a blank solution at 550 nm using a UV/VIS spectrometer Lambda 650 (Perkin Elmer, Inc., Waltham, MA, USA). Each extract was analyzed in triplicate, and assay results were expressed as a percentage per oven-dried (o.d.) extract. The confidence interval (CI) for the results did not exceed 3% at α = 0.05.

#### 3.2.3. UHPLC-ESI-MS/MS Qualitative Analysis

The identification of compounds was performed by an Acquity UPLC system (Waters Corp., Milford, MA, USA) coupled with a quadrupole-time of flight (Q-TOF) MS instrument (UPLC/Synapt Q-TOF MS, Waters, Milford, MA, USA) with an electrospray ionization (ESI) source. The UHPLC separation was carried out using a Waters Acquity BEHC18 (2.1 mm × 50 mm i.d., 1.7 µm). The mobile phase consisted of 0.1% formic acid, water (A), and acetonitrile (B), with a flow rate of 0.35 mL/min under the gradient program of 5–20% (B) for an initial 1 min, 20–25% (B); 5–6 min, 25–75% (B), 6–7 min, 75–80% (B), 7–8 min, 80–5% (B), 8–10 min, 5% (B); the injection volume was 2.0 μL.

Mass spectrometric analysis was conducted in negative and positive ion mode, and the full scan mass spectral data were collected over a range from *m*/*z* 50 to 1200. The optimum source parameters were as follows: capillary voltage, 2.5 kV (−); cone voltage, 60 V; cone gas flow, 50 L/h; collision energy, 6 eV; source temperature, 120 °C; desolvation temperature, 350 °C; collision gas, argon; desolvation gas, nitrogen; flow rate, 500 L/h.

#### 3.2.4. Purification of PACs

The purification of PACs from non-tannin and sugar was carried out using a solvent-resistant (SR) column packed with Sephadex LH-20 with 96% EtOH and 70% (*v*/*v*) acetone as the respective purification solvents. In the purification process, low-molecular-weight phenolics were eluted with 96% EtOH until the absorbance at 280 nm started to approach zero, and the PACs were eluted with 70% (*v*/*v*) acetone. Purified CTs were evaporated using a rotary evaporator (Heidolph Instruments, Schwabach, Germany) prior to being freeze-dried and stored at −8 °C.

#### 3.2.5. Determination of the Anti-Microbial Activity

**Figure 12:**
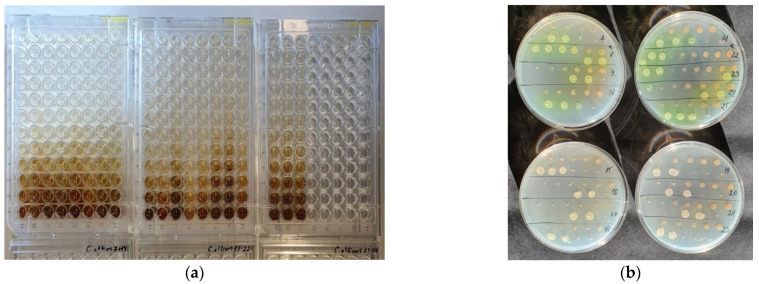
(**a**) Minimum inhibitory concentration (MIC) of ‘Maria Bruvele’ extract against *C. albicans* in 96-well plates by the two-fold serial broth microdilution method; (**b**) Minimum bactericidal concentration (MBC) of ‘Maria Bruvele’ extract against *P. aeruginosa* and minimum fungicidal concentration (MFC) against *C. albicans*.

The anti-microbial activity tests of the extracts from the twigs of 8 cultivars of SBT, purified PACs from 50% EtOH extract of ‘Maria Bruvele’ twigs, and admixture after PAC purification were performed at the Faculty of Biology, University of Latvia. To determine anti-microbial activity, several reference microbial strains, received from the Microbial Strain Collection of Latvia (MSCL), University of Latvia, were used: *Pseudomonas aeruginosa* MSCL 334, *Staphylococcus aureus* MSCL 330, *Escherichia coli* MSCL 332, *Bacillus cereus* MSCL 330, and *Candida albicans* MSCL 378. The evaluation of the anti-microbial activity of the samples against the test cultures of microorganisms was carried out according to the method for determining the sensitivity of microorganisms to anti-microbial drugs. Anti-microbial activity was studied in 96-well plates by the two-fold serial broth microdilution method, which allowed the determination of the minimum inhibitory (MIC) and minimum bactericidal/fungicidal concentrations (MBC/MFC) (Figure 12).

The MIC was determined as the lowest concentration of the studied material, which showed no visible growth.

#### 3.2.6. Cell Lines and Cultivation

The BALB/c 3T3 murine fibroblast cell line was obtained from ATCC (American Type Culture Collection, Manassas, VA, USA). Cells were propagated in DMEM medium (Sigma, Irvine, UK) supplemented with 1% penicillin (100 U/mL)–streptomycin (100 μg/mL) and 10% calf serum (Sigma, St. Louis, MO, USA). All cultivations were performed in a humidified 5% CO_2_ atmosphere at 37 °C.

#### 3.2.7. Hemolysis Assay

A hemolysis test was performed to assess the hemocompatibility of the extracts. Blood from healthy donors was collected in Monovette vacutainers containing Ethylenediamine tetra-acetic acid (EDTA). Blood was diluted with 0.9% sodium chloride solution (4:5 ratio by volume). Extracts were added to 15 mL tubes containing fresh 9.8 mL PBS and incubated at 37 °C and 5% CO_2_ for 30 min, and 0.2 mL of diluted blood was added to each tube and incubated at 37 °C and 5% CO_2_ for 1 h and 8 h. PBS was used as a negative control and deionized water as a positive control. After incubation tubes were centrifuged at 2000 rpm for 5 min, the supernatants were collected, and the absorbance was measured at a wavelength of 545 nm in a microplate reader Tecan Infinite^®^ 200 PRO (Tecan Group Ltd., Mannedorf, Switzerland).

The hemolytic ratio (HR) was calculated by the following equation:

$$\mathrm{HR}\;(\%)=\frac{\left(\mathrm{Abs}\left(\mathrm{sample}\right)-\mathrm{Abs}\left(\mathrm{negative}\;\mathrm{control}\right)\right)}{(\mathrm{Abs}\;(\mathrm{positive}\;\mathrm{control})-\mathrm{Abs}\left(\mathrm{negative}\;\mathrm{control}\right))}\;\times \;100$$

#### 3.2.8. Cytotoxicity Assay

The cytotoxicity of the extracts was tested for the BALB/c3T3 cell line by the neutral red (NR) uptake assay. Cells were seeded in 96-well plates at a density of 5 × 10^3^ cells per well. After 24 h of incubation, extracts in a concentration range of 0.125 to 4 mg/mL were added. Dilutions were made in a cell cultivation medium. Cultivation in the presence of extracts was performed for 48 h. Afterward, the plates were washed with phosphate-buffered saline (PBS) (Sigma, D8537, Irvine, UK), and a 25 µg/mL NR solution (Sigma, N2889, Irvine, UK) diluted in 5%-fetal-calf-serum-containing-media was added. After 3 h incubation in a humidified 5% CO_2_ atmosphere at 37 °C, the plate was washed with PBS, and the NR taken up by viable cells was extracted using desorbing fixative (50% ethanol/1% acetic acid/49% water). Absorbance at 540 nm was measured using a microplate reader Tecan Infinite^®^ 200 PRO (Tecan Group Ltd., Mannedorf, Switzerland). Cytotoxicity was expressed as a concentration-dependent reduction in the uptake of NR, compared to the untreated controls.

#### 3.2.9. Quantification of IL-8 and IL-6 Release from Human Peripheral Blood Mononuclear Cells (PBMNCs)

The effect of the extracts was evaluated in human peripheral blood mononuclear cells (PBMNCs). Blood from healthy donors was collected in Monovette vacutainers containing EDTA. Blood was collected in accordance with the approval of the Committee of Research Ethics of the Institute of Cardiology and Regenerative Medicine, University of Latvia. Blood was diluted (1:2 ratio by volume) with 0.9% sodium chloride solution supplemented with 10 U/mL heparin and mononuclear cell fraction isolated by gradient centrifugation. Diluted blood samples were layered on Ficoll-Paque solution (GE Healthcare, Chicago, IL, USA), and density gradient centrifugation was performed at 800× *g* for 20 min at room temperature in a swing-out centrifuge. Mononuclear cells containing buffy coats were aspirated and washed twice with phosphate-buffered saline and centrifuged at 600× *g* for 20 min at room temperature. The cell pellet was suspended in DMEM medium (Sigma, D6046, Irvine, UK) supplemented with 1% penicillin (100 U/mL)–streptomycin (100 μg/mL) and 10% fetal bovine serum (Sigma, St. Louis, MO, USA), and cells were seeded on 24-well plates at a density of 3 × 10^5^ cells per well and incubated at 37 °C, 5% CO_2_. Cells were allowed to adhere overnight prior to the addition of extracts at concentrations 0.5 and 0.25 mg/mL, 10 µg/mL poly I:C (Sigma, St. Louis, MO, USA), or a combination of both. Cells were incubated for 4 or 24 h at 37 °C, 5% CO_2_, and incubation media were collected and stored at −80 °C for further analysis.

Concentrations of IL-8 or IL-6 secreted in cultivation media by PBMNCs were determined using enzyme-linked immunosorbent assay (ELISA). Human IL-8 DuoSet ELISA kits (RnD Systems^®^, Minneapolis, MN, USA) were used according to the manufacturer’s recommendations.

### 3.3. Statistical Analysis

All measurements were conducted in triplicate, and the results are presented as the mean value ± standard deviation (SD). Statistical analyses were performed using Microsoft Excel 2016. Confidence intervals for a mean using Student’s T distribution were calculated at a significance level α = 0.05. A significance level of *p* < 0.05 was used.

For quantification of IL-8 and IL-6 release from human PBMNCs, the data were analyzed and graphs were generated using GraphPad Prism 5.0 software (San Diego, CA, USA). One-way ANOVA test was used. Differences were considered statistically significant if *p* < 0.01 and *p* < 0.05 (corresponding level was marked on the Figures).

## 4. Conclusions

The study showed that lignocellulosic biomass after harvesting, particularly, SBT twigs, could be a potential source of anti-inflammatory, anti-bacterial, and anti-fungal treatments. The 50% EtOH extracts have higher anti-bacterial and anti-fungal properties and between all the cultivar extracts, ‘Maria Bruvele’ is one of the most prospective sources for anti-bacterial treatments, while the extracts from ‘Tarmo’ are the most prospective for anti-fungal treatments. PACs isolated from SBT twigs have much higher anti-bacterial and anti-fungal properties, and in addition, they have high anti-inflammatory activity. Therefore, PACs having all these valuable properties in complex, not only inhibiting the pathogen by itself but also suppressing the inflammation that it provokes, are show very good prospects as a new therapeutic agent in prophylaxis and treatment of bacterial and fungal infections. Due to the low cytotoxicity in the bacteria/fungi inhibition diapason, PACs could also be studied for the treatment of internal organ infections. It should be noted that to align with 3R principles (replacement, reductions, refinement), in vitro testing is crucial—it allows the selection of the most prospective samples and provides information about the starting doses, resulting in a reduced number of animals needed for such studies. This study has produced a valuable volume of data for the later follow-up with in vivo testing.

## Figures and Tables

**Table 1:** Tentatively identified components in tested SBT extracts.

Peak No.	t_R_ (min)	[M−H]^−^(*m*/*z*)	Fragments	Identification
1	0.41	341.1124	179; 161; 143; 119; 113; 101	Sucrose, fructose, glucose
2	0.47	191.0239	111; 173; 127; 85	Quinic acid
3	0.98	175.0778	159; 147	Serotonin
4	1.84	305.0706	179; 125	Gallocatechin or its isomer epigallocatechin
5	1.89	593.1289	407; 425; 305; 467; 289	(epi)catechin-(epi)gallocatechin
6	1.97	1185.2393	881; 593; 305; 289; 245	Procyanidin tetramer
7	2.06	1055.2609	881; 593; 305; 289	Procyanidin tetramer
8	2.30	865.1929	577; 289; 245	Procyanidin trimer
9	2.38	289.0754	245; 125	Catechin/Epicatechin
10	2.50	1153.2501	865; 577; 289; 245	Procyanidin tetramer
11	3.28	609.4297	301; 271	Quercetin-3-O-rutinoside
12	3.33	301.0027	286; 109	Quercetin
13	7.14	487.3439	293; 117	Triterpenoid
14	7.79	471.3486	452; 265; 117	Triterpenoid
15	7.86	471.3490	265; 117	Triterpenoid
16	8.07	455.3535	277; 117	Triterpenoid
17	8.01	617.3828	255; 117	Acylated triterpenoid

**Table 2:** Anti-bacterial and ant—fungal activity of the extracts from SBT samples.

SBT Cultivars	*E.coli* MIC/MBC, mg/mL	*P. aeruginosa* MIC/MBC, mg/mL	*S. aureus* MIC/MBC, mg/mL	*B. cereus* MIC/MBC, mg/mL	*C. albicans* MIC/MFC, mg/mL
	50% EtOH extracts				
Maria Bruvele	0.2/0.2	0.39/0.78	0.2/0.39	0.39/50	0.2/>50
Bot. Lub.	0.39/0.39	0.78/1.56	0.39/0.78	0.78/50	0.2/>50
Tatiana	0.39/0.39	3.13/3.13	0.2/0.78	0.78/50	0.39/>50
Leikora	0.39/0.39	0.78/1.56	0.39/0.78	0.39/12.5	12.5/25
Duet	0.2/0.2	0.78/0.78	0.39/0.78	0.39/12.5	12.5/25
Otto	0.2/0.2	0.78 /1.56	0.39/0.78	0.39/12.5	6.25/25
Clara	0.2/0.2	0.78/1.56	1.56/3.13	0.39/12.5	12.5/25
Tarmo	0.78/0.78	0.78/1.56	0.78/0.78	0.39/12.5	6.25/12.5
	Water extracts				
Maria Bruvele	0.39/0.39	0.39/3.13	0.39/0.78	0.78/>50	0.39/>50
Bot. Lub.	0.78/50	0.78/50	0.39/12.2	0.78/>50	0.39/>50
Tatiana	0.39/0.39	0.78/1.56	0.39/0.78	0.78/>50	0.39/>50
Leikora	0.39/0.39	1.56/1.56	1.56/1.56	0.78/25	12.5/12.5
Duet	0.39/>50	1.56/>50	12.5/12.5	1.56/25	12.5/25
Otto	0.78/>50	6.25/50	6.25/12.5	0.78/25	12.5/25
Clara	0.39/0.39	1.56/1.56	0.78/0.78	0.78/25	12.5/25
Tarmo	0.39/0.39	0.78/1.56	0.78/1.56	0.78/25	12.5/12.5

**Table 3:** PACs’ role in anti-microbial activity against *B. cereus* and *C.albicans*.

Samples	*B. cereus* MIC/MBC, mg/mL	*C.albicans* MIC/MFC, mg/mL
Maria Bruvele 50% EtOH extract	0.39/50	0.20/>50
Maria Bruvele water extract	0.78/>50	0.39/>50
PACs	0.63/1.25	1.25/>2.5
Impurities	1.56/>50	12.5/>50

## Data Availability

Data are contained within the article.
